# Vitamin D Status and SARS-CoV-2 Infection and COVID-19 Clinical Outcomes

**DOI:** 10.3389/fpubh.2021.736665

**Published:** 2021-12-22

**Authors:** Iacopo Chiodini, Davide Gatti, Davide Soranna, Daniela Merlotti, Christian Mingiano, Angelo Fassio, Giovanni Adami, Alberto Falchetti, Cristina Eller-Vainicher, Maurizio Rossini, Luca Persani, Antonella Zambon, Luigi Gennari

**Affiliations:** ^1^Department of Endocrine and Metabolic Diseases, Istituto di Ricovero e Cura a Caratttere Scientifico (IRCCS), Istituto Auxologico Italiano, Milan, Italy; ^2^Department of Medical Biotechnology and Translational Medicine, University of Milan, Milan, Italy; ^3^Rheumatology Unit, University of Verona, Verona, Italy; ^4^Biostatistic Unit, Istituto di Ricovero e Cura a Caratttere Scientifico (IRCCS), Istituto Auxologico Italiano, Milan, Italy; ^5^Department of Medicine, Surgery and Neurosciences, University of Siena, Siena, Italy; ^6^Unit of Rehabilitation Medicine, San Giuseppe Hospital, Istituto di Ricovero e Cura a Caratttere Scientifico (IRCCS), Istituto Auxologico Italiano, Piancavallo, Italy; ^7^Unit of Endocrinology, Fondazione Istituto di Ricovero e Cura a Caratttere Scientifico (IRCCS) Cà Granda, Milan, Italy; ^8^Department of Statistics and Quantitative Methods, Università di Milano-Bicocca, Milan, Italy

**Keywords:** vitamin D, COVID-19, mortality, SARS-CoV-2 infection, respiratory distress syndrome, intensive care unit

## Abstract

**Background:** Several studies suggest an association between serum 25-hydroxyvitamin D (25OHD) and the outcomes of Severe Acute Respiratory Syndrome Corona-Virus-2 (SARS-CoV-2) infection, in particular Coronavirus Disease-2019 (COVID-19) related severity and mortality. The aim of the present meta-analysis was to investigate whether vitamin D status is associated with the COVID-19 severity, defined as ARDS requiring admission to intensive care unit (ICU) or mortality (primary endpoints) and with the susceptibility to SARS-CoV-2 and COVID-19-related hospitalization (secondary endpoints).

**Methods:** A search in PubMed, ScienceDirect, Web of Science, Google Scholar, Scopus, and preprints repositories was performed until March 31th 2021 to identify all original observational studies reporting association measures, or enough data to calculate them, between Vitamin D status (insufficiency <75, deficiency <50, or severe deficiency <25 nmol/L) and risk of SARS-CoV-2 infection, COVID-19 hospitalization, ICU admission, or death during COVID-19 hospitalization.

**Findings:** Fifty-four studies (49 as fully-printed and 5 as pre-print publications) were included for a total of 1,403,715 individuals. The association between vitamin D status and SARS-CoV2 infection, COVID-19 related hospitalization, COVID-19 related ICU admission, and COVID-19 related mortality was reported in 17, 9, 27, and 35 studies, respectively. Severe deficiency, deficiency and insufficiency of vitamin D were all associated with ICU admission (odds ratio [OR], 95% confidence intervals [95%CIs]: 2.63, 1.45–4.77; 2.16, 1.43–3.26; 2.83, 1.74–4.61, respectively), mortality (OR, 95%CIs: 2.60, 1.93–3.49; 1.84, 1.26–2.69; 4.15, 1.76–9.77, respectively), SARS-CoV-2 infection (OR, 95%CIs: 1.68, 1.32–2.13; 1.83, 1.43–2.33; 1.49, 1.16–1.91, respectively) and COVID-19 hospitalization (OR, 95%CIs 2.51, 1.63–3.85; 2.38, 1.56–3.63; 1.82, 1.43–2.33). Considering specific subgroups (i.e., Caucasian patients, high quality studies, and studies reporting adjusted association estimates) the results of primary endpoints did not change.

**Interpretations:** Patients with low vitamin D levels present an increased risk of ARDS requiring admission to intensive care unit (ICU) or mortality due to SARS-CoV-2 infection and a higher susceptibility to SARS-CoV-2 infection and related hospitalization.

## Introduction

Coronavirus Disease-2019 (COVID-19) is a rapidly evolving pandemic disease due to the infection by Severe Acute Respiratory Syndrome Corona-Virus-2 (SARS-CoV-2), the seventh coronavirus able to infect humans. The virus has high transmissibility, mainly through droplets issued with the phonation and breath or by direct contact (1, 2). The clinical expression of the disease ranges from completely asymptomatic forms up to dramatic pictures such as the Acute Respiratory Distress Syndrome (ARDS) often associated with multi-organ failure (1–3). Pending targeted antiviral approaches, the current clinical management is essentially based on the control of the abnormal inflammatory response and respiratory support in a hospital setting. To date, there is limited information available for effective treatment, and concerning the factors that can affect susceptibility to infection and its severity.

Especially in the first phase of the pandemic, SARS-CoV-2 infection was more frequent and aggressive, particularly considering mortality rate, in the Southern European countries (e.g., Italy and Spain), where hypovitaminosis D is most common (4, 5). The elderly, and particularly those living in the care homes, are those who have paid the most dramatic consequences of the disease. As known, they also represent a population in which hypovitaminosis D is substantially endemic. Similar considerations could be made regarding obese subjects or individuals with darker skin (6). Above all, COVID-19 importantly affects the respiratory tract, and the data supporting a significant effect of vitamin D in preventing and mitigating respiratory tract infections have emerged. A meta-analysis of individual participant data published in 2017 analyzed over 11,300 patients from 25 randomized studies and demonstrated the protective effect of vitamin supplementation D on respiratory tract infections. The effect was not only statistically significant but also clinically relevant, with a Number Needed to Treat (NNT) of 33. This already positive result appeared even more impressive in the subgroup of subjects with severe vitamin D deficiency (NNT = 8) (7). The latter observation is of relevance, given that most of the latest clinical megatrials on vitamin D shared the serious limitation of having enrolled a large proportion of not vitamin D deficient subjects, and often even with values well above the ideal limit (7). However, it should be noted that COVID-19 not only affects the respiratory tract but can also cause acute cardiac injury, acute kidney injury, acute liver injury, sepsis, and shock (3).

A few years ago, the European Society for Clinical and Osteoarthritis (ESCEO) stated that there was not enough evidence to recommend the use of vitamin D supplementation for the prevention and/or the treatment of extra-skeletal conditions. However, this same position paper underlined the growing amount of data suggesting its possible extra-skeletal effects (8). More recently, an evidence-based report from the Scientific Advisory Committee on Nutrition (SACN) of the UK concluded that there may be some benefit from daily, low-dose vitamin D supplementation in reducing risk of acute respiratory tract infections (9). This has been further emphasized by an updated meta-analysis of 46 eligible randomized controlled trials on vitamin D supplementation for the prevention of acute respiratory tract infections (10).

The immunomodulatory role of vitamin D is also well-known (11). It supports the innate immunity through the production of several antimicrobial peptides (cathelicidins, defensivins, and IL-37). Furthermore, vitamin D acts on adaptive immunity by modulating the main proinflammatory cytokines (e.g., IL-6, TNF-alpha, and interferon-gamma) and controlling the response mediated by Th1 lymphocytes (11, 12). This regulation is expected to be less efficient in conditions of vitamin D deficiency, but could be restored after adequate supplementation.

A preliminary study that evaluated the antiviral potential of various molecules against SARS-CoV-2 documented the inhibitory effect of calcitriol (1,25-dihydroxyvitamin D), the active vitamin D metabolite, on the nasal epithelium infected with the virus (13). Furthermore, vitamin D replacement intervention has been suggested to prevent the risk of respiratory failure in patients with SARS-CoV-2 infection, in some but not all observational or randomized studies (14–16). Together with these observations, there is a growing number of data showing an association between serum 25-hydroxyvitamin D (25OHD), the widely accepted marker of vitamin D status and the different clinical outcomes of SARS-CoV-2 infection, particularly concerning COVID-19 related severity and mortality, as further underlined by some pilot meta-analysis studies (17–19). Since the publication of these studies, several new data have been released, that have cast some light even on the 25OHD thresholds defining vitamin D status possibly associated with the SARS-CoV-2 infection susceptibility and COVID-19 related outcomes.

Given the current lack of specific treatment and the severe health and economic burden of the pandemic, it is of paramount importance to investigate the risk factors for the transmission of SARS-CoV-2 infection and the clinical course of COVID-19, especially until an effective vaccine strategy becomes available on a large scale. Likewise, transparency and rapid communication appear of major relevance at this stage (20). In this scenario, vitamin D deficiency may represent an easily modifiable risk factor, particularly considering the limited cost and the safety of vitamin D supplementation. Therefore, the primary end-point of the present systematic review and meta-analysis was to synthetize the currently available evidence on the role of vitamin D status as predictor of COVID-19 severity [i.e., requiring admission to Intensive Care Unit (ICU) or impacting on mortality in hospitalized patients]. The secondary endpoint was to assess the evidence of the association between vitamin D status and the susceptibility to SARS-Cov2 infection, or COVID-19-related hospitalization. Importantly, since most studies measured 25OHD levels at the time Sars-CoV-2 infection, they cannot completely rule out the possibility of reverse causality, particularly concerning the secondary endpoints, i.e., that the acute illness could have led to a reduction in total 25OHD levels, as suggested by previous observations (21, 22). Thus, we also separately considered studies that used 25OHD levels measured before infection from those that assessed 25OHD status at the time of COVID-19 diagnosis and/or hospitalization.

## Methods

### Search Strategy and Eligibility Criteria

The PRISMA guidelines have been followed for carrying out the meta-analysis (23). We searched PubMed, ScienceDirect, Web of Science, Scopus, Google Scholar, and preprints repositories (i.e., ^*^MedXriv, Research square, Social Science Research Network) until March 31th 2021, by using the following terms: ((“COVID 19”[All Fields] OR “COVID 19”[MeSH Terms] OR “COVID 19 vaccines”[All Fields] OR “COVID 19 vaccines”[MeSH Terms] OR “COVID 19 serotherapy”[All Fields] OR “COVID 19 serotherapy”[Supplementary Concept] OR “COVID 19 nucleic acid testing”[All Fields] OR “COVID 19 nucleic acid testing”[MeSH Terms] OR “COVID 19 serological testing”[All Fields] OR “COVID 19 serological testing”[MeSH Terms] OR “COVID 19 testing”[All Fields] OR “COVID 19 testing”[MeSH Terms] OR “sars cov 2”[All Fields] OR “sars cov 2”[MeSH Terms] OR “severe acute respiratory syndrome coronavirus 2”[All Fields] OR “ncov”[All Fields] OR “2019 ncov”[All Fields] OR ((“coronavirus”[MeSH Terms] OR “coronavirus”[All Fields] OR “cov”[All Fields]) AND 2019/11/01:3000/12/31[Date—Publication])) AND (“vitamin d”[MeSH Terms] OR “vitamin d”[All Fields] OR “ergocalciferols”[MeSH Terms] OR “ergocalciferols”[All Fields])) OR ((“sars cov 2”[MeSH Terms] OR “sars cov 2”[All Fields] OR “sars cov 2”[All Fields]) AND (“vitamin d”[MeSH Terms] OR “vitamin d”[All Fields] OR “ergocalciferols”[MeSH Terms] OR “ergocalciferols”[All Fields])). The Mendeley Desktop application (version 1.18, Mendeley Ltd.) was used to remove the duplicates and apply the inclusion criteria. No language or publication status limits were applied.

We included all original studies (excluding case reports, review articles, editorial, meta-analyses) recruiting COVID-19 hospitalized patients (with information about ICU admission and/or death) or patients with and without SARS-CoV-2 infection (from both serological and naso-pharyngeal swab, associated with reverse transcription-polymerase chain reaction analysis) or COVID-19 patients requiring and not requiring hospitalization and reporting prevalence of vitamin D insufficiency (25OHD levels <75 ml/L) and/or vitamin D deficiency (25OHD levels <50 nmol/L) and/or severe vitamin D deficiency (25OHD levels <25 nmol/L)0.1 (2). For this latter threshold, we considered together studies reporting data of patients with 25OHD levels <25 and <20 nmol/L, as both these cut-offs are generally considered linked with severe vitamin D deficiency (24). The choice of considering all these 25OHD levels thresholds is related to the fact that nowadays a wide agreement on which would be the most reliable threshold for defining normal 25OHD levels is still lacking (24, 25).

In this meta-analysis we considered as primary endpoint the COVID-19 severity outcome defined as ARDS requiring admission to intensive care unit (ICU) or mortality. Secondary endpoints included the susceptibility to SARS-Cov2 infection, and COVID-19-related hospitalization.

For ICU admission, given the different behavior among countries for the treatment and management of severe COVID-19 patients during the SARS-Cov2 pandemic, also including different clinical approaches to patients with ARDS, we joined the studies clearly defining the group of subjects in ICU with those reporting data from patients treated with continuous positive airway pressure (CPAP) and/or endo-tracheal intubation, even in the absence of a clear statement of ICU admission.

### Selection Studies and Data Extraction

Two authors (IC and LG) independently screened titles and abstracts and reviewed the full text of potentially relevant studies. They discussed questionable studies to agree on their possible inclusion in the present analysis.

The following data were extracted from the included studies: authors, publication form (full print or pre-print), study location, period of the year, data collection and study design, sample size, mean age, percentage of male patients, time of 25OHD measurement (before infection or at COVID-19 diagnosis), methods of 25OHD measurement (immune-assays or mass spectrometry), 25OHD threshold(s), ethnicity, study outcome (i.e., infection, hospitalization, severity, and death), prevalence of major comorbidities (when available), association estimate (Odds Ratio—OR) and relative 95% Confidence Intervals (95% CIs), use of adjustment approach for the association estimate. When adjusted or raw association estimate was not available, OR with 95% CI were calculated from the proportions of patients with outcome in each category defined by 25OHD thresholds for, respectively, insufficiency, deficiency and severe deficiency, assuming asymptotic normal distribution of log OR. In the presence of studies with zero-cell counts we added a fixed value equal to 0.5 to all cells of the study to estimate the raw OR^1^.

The same investigators independently assessed the quality of the included studies using the Newcastle-Ottawa scale (NOS) (26). Discrepancies were discussed among all the Authors and resolved by consensus.

### Statistical Analysis

For each serum 25OHD threshold defining vitamin D status and each outcome (24), a pooled OR and the corresponding 95% CI was obtained according to the random effect method proposed by Der Simonian and Laird (27). Since most studies measured 25OHD levels at the time SARS-CoV-2 infection, they cannot completely rule out the possibility of reverse causality, particularly concerning the secondary endpoints, i.e., that the acute illness could have led to a reduction in total 25OHD levels (21, 22). Thus, we also estimated a pooled OR separately for studies using 25OHD levels measured before infection and for those that assessed 25OHD status at the time of COVID-19 diagnosis and/or hospitalization.

Statistical heterogeneity among association estimates was evaluated by means of *I*^2^ of Higgins, varying from 0 to 100%. Values of this index >75% suggest high heterogeneity (28).

Moreover, diagnostic and sensitivity analyses were performed for both primary endpoints. We implemented an influence analysis to investigate the impact of each study-specific association estimate on the pooled OR and a visual inspection of the funnel plot, as well as the Egger's test estimation, to evaluate publication bias presence (29). To verify the robustness of findings, we estimated the pooled ORs in selected studies with: i) high quality (NOS score ≥ 7), ii) high quality (NOS score ≥ 7) and performed in white Caucasian patients only, and (iii) high quality (NOS score ≥ 7) and reporting adjusted association estimates. Moreover, when possible, we explored changes of findings in relation to specified age categories (above or below 65 yrs), male prevalence (above or below 60%), hypertension prevalence (above or below 45%) and diabetes prevalence (above or below 25%).

Results were considered statistically significant when two-tailed *p*-value was lower than 0.05. All analyses were performed with R version 4.0.3 (R Foundation for Statistical Computing, Vienna, Austria).

## Results

### Study Selection Process

**Figure 1:**
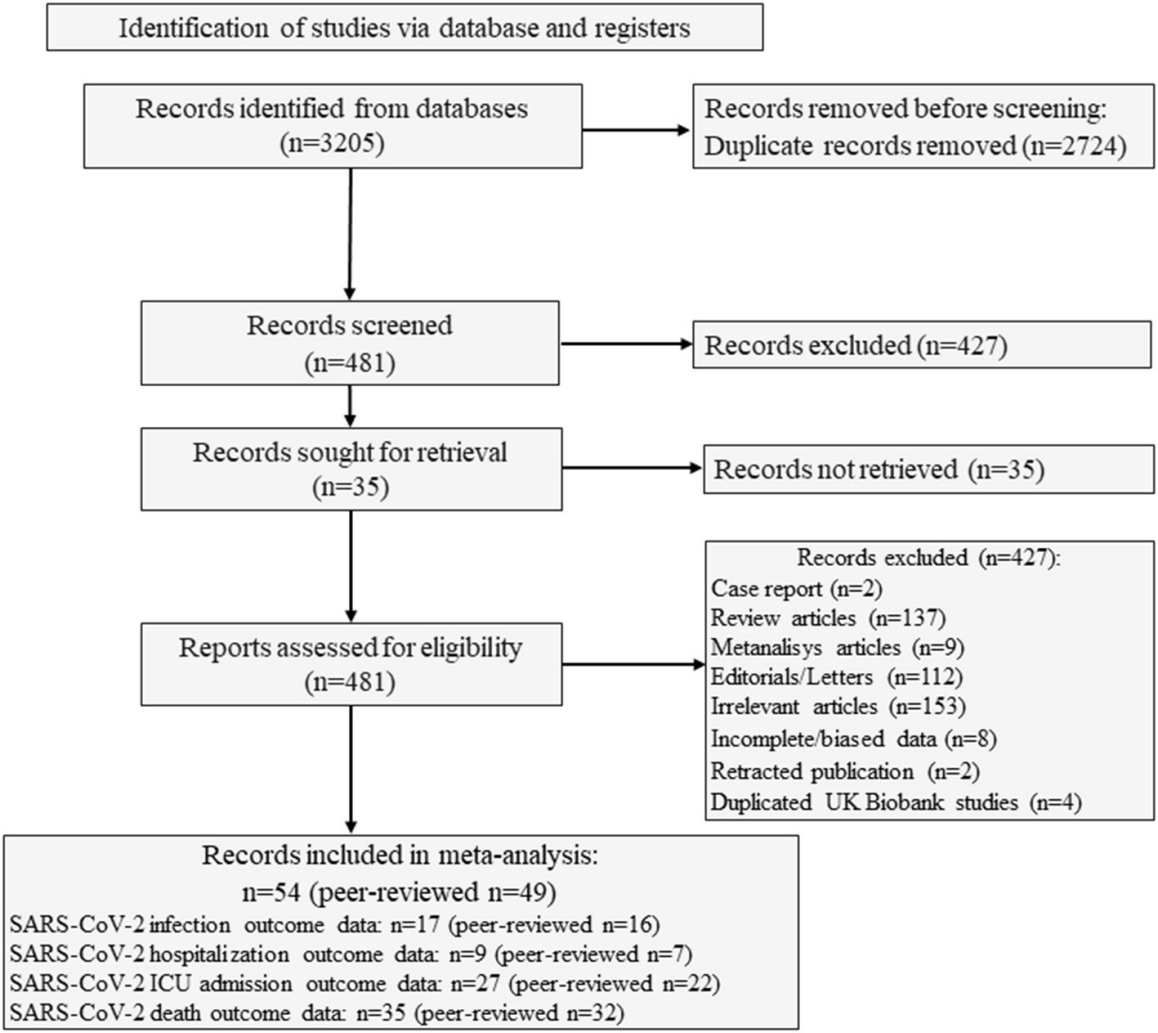
Flow of study selection process. SARS-CoV-2, Severe Acute Respiratory Syndrome Corona-Virus-2.

The study selection process is summarized in Figure 1. We identified 3,205 studies from the different searched databases and excluded 2,724 studies for duplication. The remaining 481 studies were first screened by reading the title and abstract. Four-hundred-thirteen studies were then excluded as they were case reports (*n* = 2), reviews (*n* = 137), meta-analysis (*n* = 9), editorial/letters (*n* = 112), or because they were not relevant for the aims of the present meta-analysis (*n* = 153). Two studies available as preprints until August 2020 were excluded since they were subsequently retracted (30–32). In addition, 7 studies were excluded due to incomplete data, mainly due to the lack of information concerning disease outcomes in relation to 25OHD thresholds (33–39) or because severe COVID-19 cases requiring intubation or mechanical respiratory support were not considered for 25OHD measurement (40). Finally, among the 5 studies based on the United Kingdom Biobank database (41–45), the most recent and updated paper was included (45), which reports updated information on 1,082 confirmed COVID19 cases with respect to the 449 cases of the previous publications. The inter-rate reliability between the two authors in the selection process was good (κ = 0.89).

Eventually, 54 studies (49 as full print and 5 as pre-print publication) were entered into the meta-analysis whose characteristics are summarized in Table 1 (45–98). Among the 54 included studies (1,403,715 individuals), data collection was retrospective for 37 studies and prospective for 17 studies. Among the 5 studies published as pre-print version, 3 were retrospective and 2 were prospective. Among the included studies, 27 reported the association between vitamin D status and COVID-19 related ICU admission (4,608 individuals), 35 reported the association between vitamin D status and COVID-19 related mortality (9,664 individuals), 17 examined the differences in vitamin D status between SARS-CoV2 positive and negative cases (1, 395, 721 individuals) and 9 considered the association between vitamin D status and COVID-19 related hospitalization (2,817 individuals). A summary of the number of studies and the sample size considered for each specific outcome in relation to the serum 25OHD thresholds of 25, 50, and 75 nmol/L is given in Table 2.

**Table 1:** Summary of characteristics and quality evaluation by Newcastle Ottawa Scale score (NOSs) of the studies included in the meta-analysis.

**Author** **[reference]**	**Country**	**Period** **(yr 2020)**	**Design**	**Time**	**VD** **<25**	**VD** **<50**	**VD** **<75**	**Meth**.	**Size** **(*n*)**	**Age** **(yrs)**	**Males** **(%)**	**Ethnicity**	**NOSs** **(0–9)**	**OR** **Adj**
**Outcome: infection**														
Abdollhai et al. (46)	Iran	Feb-Apr	Prosp	Inf	Yes	No	Yes	n.a.	402	48.0	68	Iranian	6	No
Alguwaihes et al. (47)	Saudi Arabia	May-Jul	Retr	Inf	Yes	Yes	No	IA	222	55.5	65	51% Saudi	5	Yes
Brandao et al. (48)	Brazil	Mar-Jul	Retr	Inf	Yes	Yes	Yes	IA	13,930	n.a.	n.a.	Mixed	4	No
Campi et al. (49)	Italy	Mar-Apr	Prosp	Inf	Yes	Yes	Yes	IA	361	65.3	65	Caucasian	9	Yes
Chang et al. (50)	USA	Mar-Jun	Retr	Bef	Yes	No	No	n.a.	26,602	44.0	49	Mixed	7	No
Demir et al. (51)	Turkey	Mar-Nov	Retr	Bef	Yes	Yes	Yes	n.a.	487	45.0	43	n.a. (likely Caucasian)	7	No
De Smet et al. (52)	Belgium	Mar-Apr	Retr	Inf	No	Yes	Yes	MS	2,903	69.0^*^	55	Caucasian	9	Yes
Faniyi et al. (53)	UK	Mar-Apr	Prosp	Inf	Yes	No	No	MS	392	41.0^*^	26	71% Caucasian	6	Yes
Ferrari and Locatelli (54)	Italy	Mar-Apr	Retr	Inf	No	No	Yes	IA	347	61.0	55	n.a. (likely Caucasian)	6	No
Hernandez et al. (55)	Spain	Mar-Mar	Retr	Inf	No	Yes	No	IA	197	61.0	62	Caucasian	9	No
Katz et al. (56)	USA	Mar-Jun	Retr	Bef	Yes	No	No	n.a.	987,849	n.a.	56	Mixed	7	Yes
Im et al. (57)	South Korea	Feb-Jun	Prosp	Inf	Yes	Yes	No	MS	200	52.2^*^	42	Asian	3	No
Li et al. (45)	UK	Mar-May	Retr	Bef	Yes	Yes	No	n.a.	353,299	n.a.	n.a.	94.6% Caucasian	9	Yes
Mardani et al. (58)	Iran	Mar	Retr	Inf	Yes	Yes	Yes	IA	123	43.3^*^	53	Indoeuropean	5	No
Meltzer et al. (59)	USA	Mar-Apr	Retr	Bef	No	Yes	No	n.a.	489	49.2	25	Multiethnic	8	Yes
Merzon et al. (60)	Israel	Feb-Apr	Retr	Inf	No	No	Yes	IA	7,807	35.6	49	n.a.	6	Yes
Ye et al. (61)	China	Feb-Mar	Retr	Inf	No	Yes	No	IA	183	43.0	37	Asian	6	Yes
**Outcome: Hospitalization**														
Basaran et al. (62)	Turkey	n.a.	Prosp	Hosp	Yes	Yes	No	IA	204	57.0	50	n.a. (likely Caucasian)	5	No
Campi et al. (49)	Italy	Mar-Apr	Prosp	Hosp	Yes	Yes	Yes	IA	155	65.3	65	Caucasian	9	Yes
Gavioli et al. (63)	USA	Mar-Apr	Retr	Bef	Yes	Yes	No	n.a.	437	67.0	48	Multiethnic	8	No
Im et al. (57)	South Korea	Feb-Jun	Prosp	Hosp	No	Yes	No	MS	50	52.2^*^	42	Asian	3	No
Macaya et al. (64)	Spain	Mar	Retr	Bef	No	Yes	No	IA	80	67.7^*^	44	Caucasian	7	yes
Maghbooli (65)	Iran	Until May	Retr	Hosp	No	No	Yes	IA	235	58.7	61	Indoeuropean	8	Yes
Mendy et al. (66)	USA	Mar-May	Retr	Bef	Yes	No	No	ICDc	689	49.5^*^	53	Multiethnic	7	Yes
Merzon et al. (60)	Israel	Feb-Apr	Retr	Hosp	No	No	Yes	IA	782	35.6	49	n.a.	6	Yes
Radujkovic et al. (67)	Germany	Mar-Jun	Prosp	Hosp	Yes	Yes	Yes	IA	185	60.0^*^	51	Caucasian	8	Yes
**Outcome: ICU admission**														
Adami et al. (68)	Italy	Mar-May	Retr	Hosp	Yes	Yes	Yes	IA	34	69.4	52	Caucasian	6	No
Angelidi et al. (69)	USA	Feb-Marc	Retr	Hosp	No	No	Yes	IA	144	66.0	44	Multiethnic	8	Yes
Baktash et al. (70)	UK	Mar-Apr	Prosp	Hosp	Yes	No	No	n.a.	70	79.5	61	>75% Caucasian	7	No
Barassi et al. (71)	Italy	Apr-May	Prosp	Hosp	Yes	Yes	Yes	IA	118	62.0	59	n.a. (likely Caucasian)	5	No
Campi et al. (49)	Italy	Mar-Apr	Prosp	Hosp	Yes	Yes	Yes	IA	103	65.3	65	Caucasian	9	Yes
Carpagnano et al. (72)	Italy	Mar-Apr	Retr	Hosp	Yes	No	No	IA	42	65.0	71	n.a. (likely Caucasian)	9	No
Cereda et al. (73)	Italy	Mar-Apr	Prosp	Hosp	No	Yes	No	IA	129	73.6	54	n.a. (likely Caucasian)	6	No
Charoenngam et al. (74)	USA	Mar-Aug	Retr	Bef	No	Yes	Yes	IA	287	61.0	53	67% African-American	8	Yes
Faul et al. (75)	Ireland	Mar	Prosp	Hosp	Yes	No	No	n.a.	33	n.a.	100	Caucasian	7	No
Hernandez et al. (55)	Spain	Mar	Retr	Hosp	No	Yes	No	IA	394	61.0	62	Caucasian	9	No
Im et al. (57)	South Korea	Feb-Jun	Prosp	Hosp	No	Yes	No	MS	50	52.2^*^	42	Asian	3	No
Jain et al. (76)	India	Jun-Jul	Prosp	Hosp	Yes	Yes	Yes	IA	154	46.1	62	Hindi	6	No
Jevalikar et al. (77)	India	Jul-Aug	Prosp	Hosp	No	Yes	No	IA	410	52.4	69	n.a. (likely Hindi)	6	No
Karonova et al. (78)	Russia	Apr-May	Retr	Hosp	No	Yes	Yes	IA	80	53.2	54	Caucasian	7	No
Lau et al. (79)	USA	Mar-Apr	Retr	Hosp	No	No	Yes	IA	20	65.2	45	75% African-American	6	No
Lohia et al. (80)	USA	Mar-June	Retr	Bef	No	Yes	No	n.a.	270	63.8	43	Multiethnic	6	No
Macaya et al. (64)	Spain	Mar	Retr	Bef	No	Yes	No	IA	80	67.7^*^	44	Caucasian	7	Yes
Maghbooli et al. (65)	Iran	Until May	Retr	Hosp	No	No	Yes	IA	235	58.7	61	Indoeuropean	8	Yes
Mendy et al. (66)	USA	Mar-May	Retr	Bef	Yes	No	No	ICDc	216	49.5^*^	53	Multiethnic	7	Yes
Orchard et al. (81)	UK	Kar-Jun	Retr	Hosp	No	Yes	No	IA	165	72.5	52	Multiethnic	7	No
Panagiotou et al. (82)	UK	Mar-May	Retr	Hosp	No	Yes	No	n.a.	134	71.6	54	Caucasian	6	No
Radujkovic et al. (67)	Germany	Mar-Jun	Prosp	Hosp	Yes	Yes	No	IA	93	60.0^*^	51	Caucasian	8	Yes
Szeto et al. (83)	USA	Feb-May	Retr	Bef	No	Yes	No	IA	93	60.0^*^	47	Multiethnic	4	Yes
Vanegas-Cedillo et al. (84)	Mexico	Mar-May	Prosp	Hosp	Yes	No	No	IA	551	51.9	64	n.a. (likely Hispanics)	8	Yes
Vashegani et al. (85)	Iran	Apr-Jun	Retr	Hosp	No	No	Yes	IA	508	56.0	52	Irano-Afganide	6	Yes
Walk (86)	The Netherlands	Mar-Apr	Prosp	Hosp	Yes	Yes	No	MS	133	68.0	69	n.a.	6	No
Ye et al. (61)	China	Feb-Mar	Retr	Hosp	No	Yes	Yes	IA	62	43.0	37	Asian	6	Yes
**Outcome: Death**														
Abrishami et al. (87)	Iran	Feb-Apr	Retr	Hosp	No	Yes	No	IA	73	55.2	46	Indoeuropean	9	Yes
Adami et al. (68)	Italy	Mar-May	Retr	Hosp	Yes	Yes	Yes	IA	34	69.4	52	Caucasian	6	No
Alguwaihes et al. (47)	Saudi Arabia	May-Jul	Retr.	Inf	Yes	Yes	No	IA	150	55.5	65	51% Saudi	5	Yes
Angelidi et al. (69)	USA	Feb-Marc	Retr	Hosp	No	No	Yes	IA	144	66.0	44	Multiethnic	8	Yes
Anjum et al. (88)	Pakistan	Mar-Jun	Prosp	Hosp	Yes	No	No	n.a.	140	42.5	59	n.a. (likely Asian)	5	No
Baktash et al. (70)	USA	Mar-Apr	Prosp	Hosp	Yes	No	No	n.a.	70	79.5	61	>75% Caucasian	7	No
Barassi et al. (71)	Italy	Apr-May	Prosp	Hosp	Yes	Yes	Yes	IA	118	62.0	59	n.a. (likely Caucasian)	5	No
Bennouar et al. (89)	Algeria	Jul-Aug	Prosp	Hosp	Yes	Yes	Yes	IA	120	62.3	69	African	8	Yes
Campi et al. (49)	Italy	Mar-Apr	Prosp	Hosp	Yes	Yes	Yes	IA	103	65.3	65	Caucasian	9	Yes
Carpagnano et al. (72)	Italy	Mar-Apr	Retr	Hosp	Yes	No	No	IA	42	65.0	71	n.a. (likely Caucasian)	9	No
Cereda et al. (73)	Switzerland	Mar-Apr	Prosp	Hosp	No	Yes	No	IA	129	73.6	54	Caucasian	6	Yes
Charoenngam et al. (74)	USA	Mar-Aug	Retr	Bef	No	Yes	Yes	IA	287	61.0	53	67% African-American	8	Yes
De Smet et al. (52)	Belgium	Mar-Apr	Retr	Hosp	No	Yes	No	MS	2,903	69.0	55	Caucasian	9	Yes
Gavioli et al. (63)	USA	Mar-Apr	Retr	Bef	Yes	Yes	No	n.a.	437	67.0	48	Multiethnic	8	No
Hars et al. (90)	Spain	Mar-Apr	Retr	Hosp	Yes	No	No	n.a.	160	85.9	40	n.a. (likely Caucasian)	8	Yes
Hernandez et al. (55)	India	Mar	Retr	Hosp	No	Yes	No	IA	197	61.0	62	Caucasian	9	No
Infante et al. (91)	Italy	Mar-Apr	Retr	Hosp	Yes	Yes	No	IA	137	67.0	65	Caucasian	8	No
Jain et al. (76)	Turkey	Jun-Jul	Retr	Hosp	No	Yes	No	IA	154	46.1	62	Hindi	6	No
Jevalikar et al. (77)	India	Jul-Aug	Prosp	Hosp	No	Yes	No	IA	410	52.4	69	n.a. (likely Hindi)	6	No
Karahan et al. (92)	Russia	Apr-May	Retr	Hosp	No	Yes	Yes	IA	149	63.5	54	n.a. (likely Caucasian)	9	No
Karonova et al. (78)	UK	Apr-May	Retr	Hosp	No	Yes	Yes	IA	80	53.2	54	n.a. (likely Caucasian)	7	No
Ling I et al. (93)	UK	Jan-Jul	Retr	Bef	Yes	No	No	ICDc	444	74.0	55	80% Caucasian	6	Yes
Ling II et al. (93)	UK	Jan-Jul	Retr	Bef	Yes	No	No	ICDc	540	70–76^*^	54	80% Caucasian	6	Yes
Lohia et al. (80)	USA	Mar-June	Retr	Bef	No	Yes	No	IA	270	63.8	43	Multiethnic	6	No
Luo et al. (94)	China	Feb-Mar	Retr	Hosp	Yes	No	No	IA	335	56.0	44	Asian	8	No
Maghbooli et al. (65)	Iran	Until May	Retr	Hosp	No	No	Yes	IA	206	58.7	61	Indoeuropean	8	Yes
Mardani et al. (58)	USA	Mar	Retr	Hosp	Yes	Yes	Yes	IA	63	43.3^*^	53	Indoeuropean	5	No
Mendy et al. (66)	USA	Mar-May	Retr	Bef	Yes	No	No	ICDc	216	49.5^*^	53	Multiethnic	7	Yes
Orchard et al. (81)	UK	Kar-Jun	Retr	Hosp	No	Yes	No	IA	165	72.5	52	Multiethnic	7	No
Radujkovic et al. (67)	Germany	Mar-Jun	Prosp	Hosp	Yes	Yes	No	IA	185	60.0^*^	51	Caucasian	8	Yes
Ricci et al. (95)	Italy	n.a.	n.a.	Hosp	Yes	No	No	n.a.	152	68.4	48	Caucasian	7	No
Szeto et al. (83)	USA	Until May	Retr	Bef	No	Yes	No	IA	93	60.0^*^	48	Multiethnic	4	Yes
Tehrani et al. (96)	IRAN	Mar-Apr	Retr	Hosp	Yes	No	Yes	n.a.	205	59.7	34	Asian	5	No
Tort et al. (97)	Mexico	Mar-Apr	Retr	Hosp	Yes	Yes	Yes	n.a.	172	51.4	74	n.a.	5	No
Vanegas-Cedillo et al. (84)	Mexico	Mar-May	Prosp	Hosp	Yes	Yes	No	IA	551	51.9	64	n.a. (likely Hispanics)	8	Yes
Vassiliou et al. (98)	Greece	Mar-Aug	Prosp	Hosp	No	Yes	No	IA	30	65.0	89	Caucasian	5	No

*VD, 25-hydroxyvitamin D levels; <25, <50, <75 nmol/L; Neth, method of VD measurement; Prosp, prospective; Retr, retrospective; Caucasian; NOSs, Newcastle Ottawa Scale score; OR Adj, studies reporting an odds ratio adjusted for confounders; Time, time at VD measurement; Inf., at SARS-CoV-2 infection diagnosis; Hosp, at hospitalization; Bef, before SARS-CoV-2 infection diagnosis or hospitalization; IA, immuno-assay; MS, mass spectrometry; ICDc, International Classification of Diseases code.*

*Ling I, I cohort; Ling II, II cohort; Infection, SARS-CoV-2 infection; Hospitalization, COVID-19 related hospitalization; ICU, need of intensive care unit admission for COVID-19 patients; Death, death due to COVID-19; n.a., not available.*

Age is expressed as mean or *median. The II cohort (validation cohort) of the study by Ling (ref #44) the age median values are related to two cohort of patients different hospitals. Jan, January; Fe, February; Mar, March; Apr, April; Jun, June; Jul, July; 25OHD <25, 25OHD levels <25 nmol/L; 25OHD <50, 25OHD levels <50 nmol/L; 25OHD <75, 25OHD levels <75 nmol/L. The studies by Mendy et al. (37) by Vanegas-Cedillo et al. (84) by Vashegani et al. (98) and by Walk (86) are in preprint form.

**Table 2:** Number of included studies and sample size considered for each specific outcome, in relation to the 25OHD thresholds of 25, 50, and 75 nmol/L.

**Outcome**	**25OHD Threshold**	**Included studies *n***	**Total subjects *n***	**Cases below the threshold *n* (%)**	**Cases meeting the outcome *n* (%)**
Sars-Cov2 positivity	25 nmol/L 50 nmol/L 75 nmol/L	11 12 8	1,383,867 380,357 26,333	75,837 (5.6)^#^ 195,755 (51.5) 18,578 (70.6)	5,224 (0.4%) 5,349 (1.4) 4,060 (15.4)
Hospitalization	25 nmol/L 50 nmol/L 75 nmol/L	5 6 4	1,670 1,111 1,357	274 (16.4) 627 (56.4) 429 (74.6)^*^	946 (50.6) 823 (74.1) 367 (63.8)^*^
ICU Admission	25 nmol/L 50 nmol/L 75 nmol/L	11 18 11	1,547 2,681 1,745	351 (22.7) 1,509 (56.3) 1,185 (67.9)	536 (34.6) 754 (28.1) 486 (29.4)
Death	25 nmol/L 50 nmol/L 75 nmol/L	21 24 12	4,265 4,364 1,676	892 (25.4)^§^ 2,357 (54.0) 1,141 (68.1)	892 (20.9) 811 (18.6) 336 (20.1)

# *calculated on 1,357,043 pts, excluding missing information on cases below 25OHD threshold of 25 nmol/L from Alguwaihes et al. (47) (total n = 222) and Chang et al. (50) (total n = 26,602).*

* *excluding missing information about the 782 Sars-Cov2 positive cases from Merzon et al. (60).*

§ *calculated on 3,511 pts, excluding missing information on cases below 25OHD threshold of 25 nmol/L from Alguwaihes et al. (47) (total COVID-19 cases n = 150), Hars et al. (90) (total COVID-19 cases n = 160), Radujkovic et al. (67) (total COVID-19 cases n = 185), and the 444 COVID-19 cases from the primary study population of Ling et al. (93)*.

The geographic areas of the included studies were Europe (*n* = 29), Middle East Asia (*n* = 7), North America (*n* = 9), South East Asia (*n* = 4), Central Asia (*n* = 1), Central America (*n* = 2), North Africa (*n* = 1), and Far East Asia (*n* = 1). The quality of included studies varied consistently, with a NOS between 3 and 9 (median score 6). The 25OHD levels have been determined: (i) before SARS-CoV-2 test in 5 studies (45, 50, 51, 56, 59) and at SARS-CoV-2 test in 12 studies (46–49, 52–55, 57, 58, 60, 61), (ii) before COVID-19 diagnosis in 3 studies (63, 64, 66) and at COVID-19 diagnosis in 6 studies, (49, 57, 60, 62, 65, 67), and (iii) before COVID-19 related hospitalization in 7 studies (63, 64, 66, 74, 80, 83, 93), and at COVID-19 related hospitalization in 35 studies (49, 52, 55, 57, 58, 61, 65, 67–73, 75–79, 81, 82, 84–92, 94–98).

### Primary Endpoint: Vitamin D Status as a Predictor of In-hospital COVID-19 Severity

The COVID-19 severity was first evaluated considering the need of ICU admission and then considering the COVID-19 related mortality.

**Figure 2:**
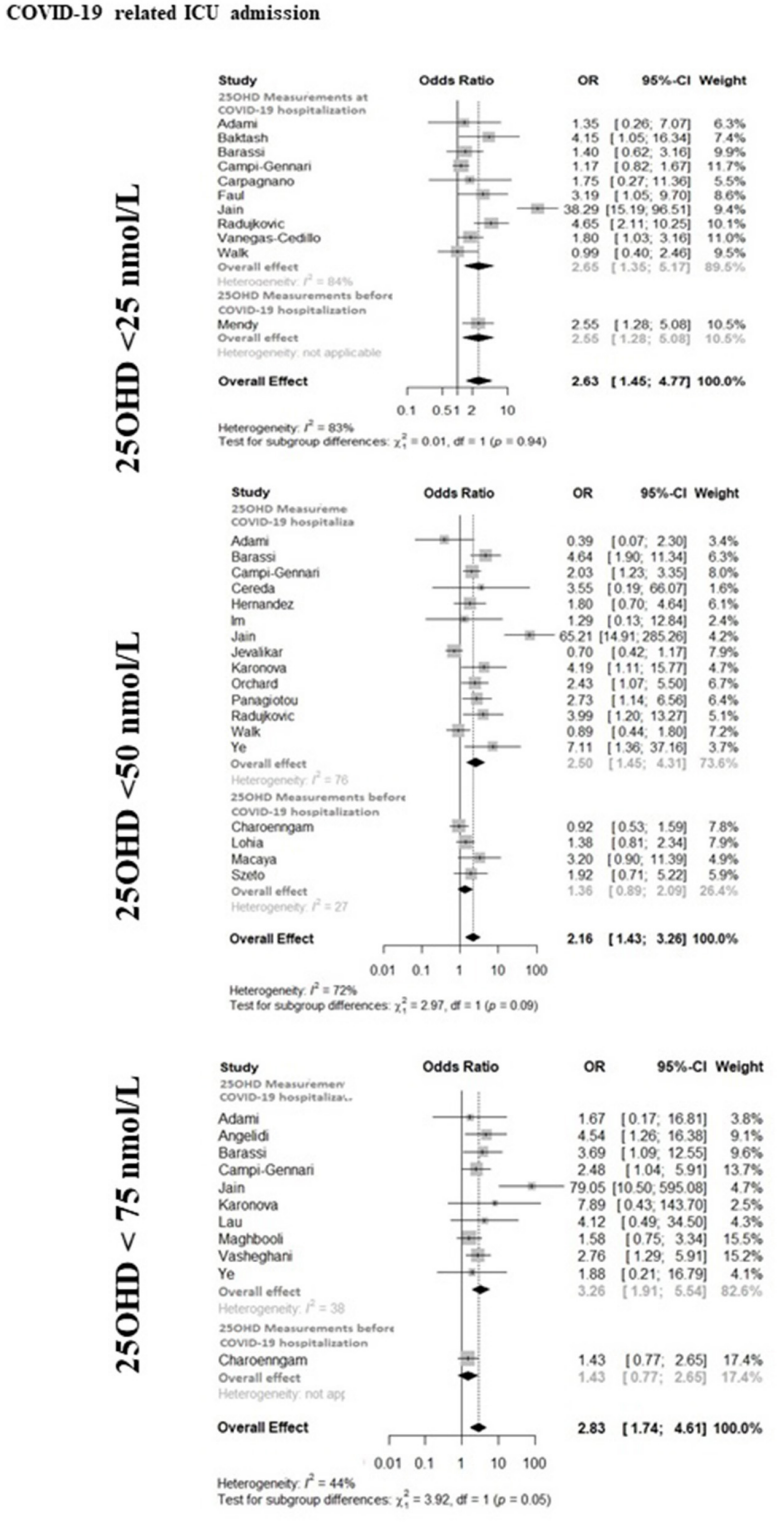
The forest-plot reporting the association between COVID-19 related admission to intensive care unit (ICU) and vitamin D thresholds (severe vitamin D deficiency, vitamin D deficiency, and vitamin D insufficiency). COVID-19, Coronavirus Disease-2019; ICU, intensive Care Unit; <25 nmol/L, 25OHD levels below 25 nmol/L; <50 nmol/L, 25OHD levels below 50 nmol/L; <75 nmol/L, 25OHD levels below 75 nmol/L.

The forest-plot reporting the association between COVID-19 related ICU admission and severe vitamin D deficiency, vitamin D deficiency or vitamin D insufficiency is shown in Figure 2. The presence of severe vitamin D deficiency (25OHD <25 nmol/L) was associated with ICU admission when considering the whole dataset (11 studies, OR 2.63, 95%CI 1.45–4.77), as well as the studies in which 25OHD levels were measured at hospitalization (10 studies, OR 2.65, 95%CI 1.35–5.17) or the only study in which 25OHD levels were measured before hospitalization (OR 2.55, 95%CI 1.28–5.08). When the 25OHD threshold of 50 nmol/L was considered, the presence of vitamin D deficiency was associated with ICU admission in the whole dataset (18 studies, OR 2.16, 95%CI 1.43–3.26), as well as in the studies in which 25OHD levels were measured at hospitalization (14 studies, OR 2.50, 95%CI 1.45–4.31). In contrast, the association did not reach the statistical significance when analyzing the studies in which 25OHD levels were measured before hospitalization (4 studies, OR 1.36, 95%CI 0.89–2.09). The presence of vitamin D insufficiency (25OHD <75 nmol/L) was associated with ICU admission when considering the whole dataset (11 studies, OR 2.83, 95%CI 1.74–4.61), as well as the studies in which 25OHD levels were measured at hospitalization (10 studies, OR 3.26, 95%CI 1.91–5.54), while did not reach the statistical significance in the only study in which 25OHD levels were measured before hospitalization (OR 1.43, 95%CI 0.77–2.65). In the latter study, however, a subgroup analysis in patients older than 65 years reported a statistically significant association between vitamin D insufficiency and the risk of ARDS or severe sepsis (74). The heterogeneity among the studies of the whole available dataset was 83, 72, and 44% for the association between the COVID-19- related ICU admission and severe vitamin D deficiency, vitamin D deficiency or vitamin D insufficiency, respectively.

**Figure 3:**
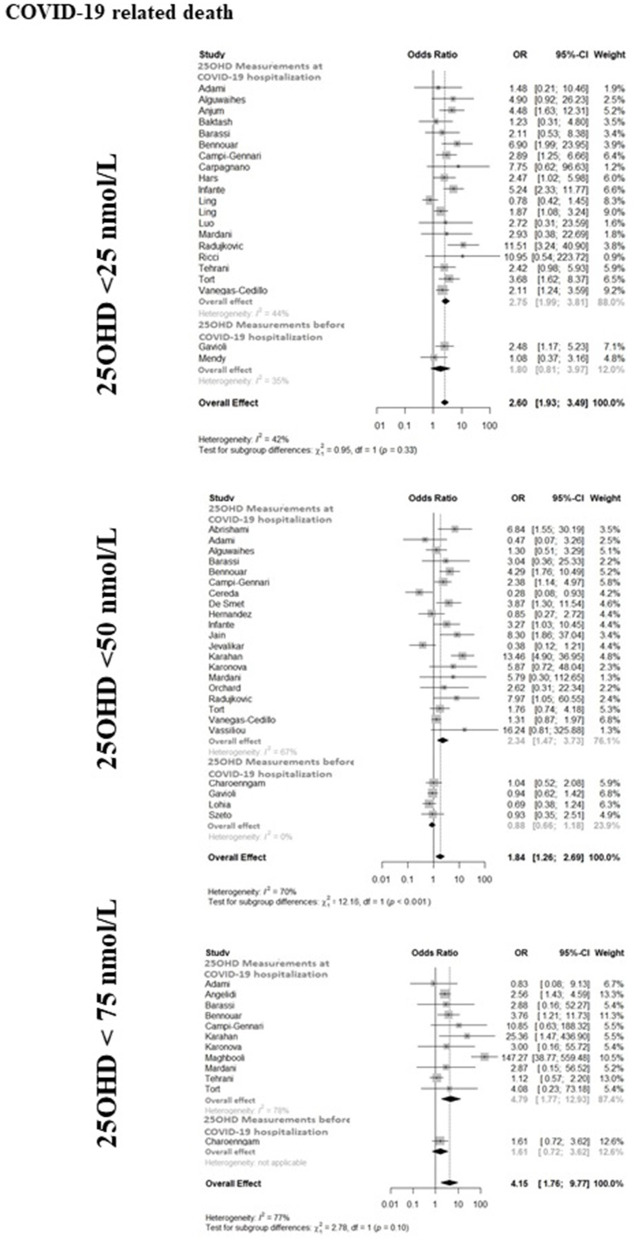
The forest-plot reporting the association between COVID-19 related mortality and vitamin D thresholds (severe vitamin D deficiency, vitamin D deficiency, and vitamin D insufficiency). COVID-19, Coronavirus Disease-2019; ICU, intensive Care Unit; <25 nmol/L, 25OHD levels below 25 nmol/L; <50 nmol/L, 25OHD levels below 50 nmol/L; <75 nmol/L, 25OHD levels below 75 nmol/L.

The forest-plot reporting the association between COVID-19 related mortality and severe vitamin D deficiency, vitamin D deficiency or vitamin D insufficiency is shown in Figure 3. The presence of severe vitamin D deficiency was associated with COVID-19 related mortality when considering the whole available dataset (21 studies, OR 2.60, 95%CI 1.93–3.49), as well as the studies in which 25OHD levels were measured at hospitalization (19 studies, OR 2.75, 95%CI 1.99–3.81). Conversely, the association did not reach the statistical significance in the studies that considered 25OHD levels measured before hospitalization (2 studies, OR 1.80, 95%CI 0.81–3.97). Likewise, the presence of vitamin D deficiency was associated with COVID-19 related mortality when considering the whole available dataset (24 studies, OR 1.84, 95%CI 1.26–2.69) or the studies in which 25OHD levels were measured at hospitalization (20 studies, OR 2.34, 95%CI 1.47–3.73), but not in the studies in which 25OHD levels were measured before hospitalization (4 studies, OR 0.89, 95%CI 0.66–1.20). The presence of vitamin D insufficiency was associated with COVID-19 related mortality when considering the whole available dataset (12 studies, OR 4.15, 95%CI 1.76–9.77), as well as the studies in which 25OHD levels were measured at hospitalization (11 studies, OR 4.79, 95%CI 1.77–12.93), while this association did not reach the statistical significance in the only study, in which 25OHD levels were measured before hospitalization (OR 1.61, 95%CI 0.72–3.62). As previously reported concerning ICU admission, the latter study described a significant association between vitamin D insufficiency and mortality in patients older than 65 years, as well as in non-obese individuals (74). The heterogeneity among the studies of whole dataset was 42, 70, and 77% for the association between the COVID-19 related mortality and severe vitamin D deficiency, vitamin D deficiency or vitamin D insufficiency, respectively.

### Secondary Endpoints: Vitamin D and SARS-CoV-2 Infection or COVID-19 Related Hospitalization

The forest-plots reporting the association between SARS-CoV-2 infection and severe vitamin D deficiency, vitamin D deficiency or vitamin D insufficiency are shown in Supplementary Figure 1. The presence of severe vitamin D deficiency or of vitamin D deficiency was associated with SARS-CoV-2 infection when considering either the whole available dataset (11 studies, OR 1.68, 95%CI 1.32–2.13 and 12 studies, OR 1.83, 95%CI 1.43–2.33, respectively), as well as the studies in which 25OHD levels were measured at the time of SARS-CoV-2 test (7 studies, OR 2.26, 95%CI 1.39–3.65 and 9 studies OR 2.17, 95%CI 1.50–3.12, respectively) or those in which 25OHD levels were measured before the SARS-CoV-2 test (4 studies, OR 1.42, 95%CI 1.09–1.84 and 3 studies OR 1.35, 95%CI 1.08–1.69, respectively). The presence of vitamin D insufficiency was associated with SARS-CoV-2 infection when considering the whole available dataset (8 studies, OR 1.49 95%CI 1.16–1.91), or the studies in which 25OHD levels were measured at the time of SARS-CoV-2 test (7 studies, OR 1.52 95%CI 1.16–1.99), but not in the only study that considered 25OHD levels measured before SARS-CoV-2 test (OR 1.22, 95%CI 0.59–2.56). The heterogeneity was relevant for almost all associations (79, 88, and 78% for the associations between SARS-CoV-2 infection and severe vitamin D deficiency, vitamin D deficiency and vitamin D insufficiency, respectively).

The forest-plot reporting the association between hospitalization due to COVID-19 and severe vitamin D deficiency, vitamin D deficiency or vitamin D insufficiency is shown in Supplementary Figure 2. The presence of severe vitamin D deficiency was associated with COVID-19 related hospitalization when considering either the whole dataset (5 studies, OR 2.51, 95%CI 1.63–3.85) or, separately, the studies in which 25OHD levels were measured before (2 studies, OR 1.99, 95%CI 1.02–3.89) or at SARS-CoV-2 test execution (3 studies, OR 3.04, 95%CI 1.46–6.33). The presence of vitamin D deficiency was associated with COVID-19 related hospitalization when considering the whole dataset (6 studies, OR 2.38 95%CI 1.56–3.63), and the studies in which 25OHD levels were measured at the time of SARS-CoV-2 test (4 studies, OR 2.84 95%CI 1.61–5.02), but not in the studies that considered 25OHD levels measured before the SARS-CoV-2 test (2 studies, OR 1.66, 95%CI 0.81–3.42). A limited number of studies (*n* = 4) investigated the relationship between vitamin D insufficiency and hospitalization following COVID-19 diagnosis, all of them assessing 25OHD at the time of SARS-CoV-2 test, with an overall OR of 1.82 (95%CI 1.43–2.33). The heterogeneity among the studies of whole dataset was 57, 60, and 0% for the association between COVID-19 related hospitalization and severe vitamin D deficiency, vitamin D deficiency or vitamin D insufficiency, respectively.

### Diagnostic and Sensitivity Analyses

The funnel plots and Egger's test *p*-value for each primary outcome and vitamin D status are reported in Supplementary Figure 3. Both did not suggest relevant publication biases except for ICU admission when serum 25OHD was <75 nmol/L threshold and for death when serum 25OHD was <50 nmol/L threshold, with a statistically significant asymmetric distribution of association estimates, suggesting high likelihood to be published for studies with overestimated risk effect. However, influence analysis did not show a relevant impact of the single study-specific association estimate on pooled ORs (Supplementary Figure 4).

The sensitivity analyses considering selected studies (i.e., only high quality studies, only high quality studies in Caucasian patients, only high quality studies reporting adjusted association estimates) did not substantially modify the findings for primary endpoints (Table 3). In particular, we observed a borderline non-significant association only for ICU and vitamin level <25 and 75 nmol/L.

**Table 3:** Pooled Odds Ratios of death or ICU admission due to COVID-19 in specific subgroups, in relation to 25OHD thresholds for vitamin D insufficiency, deficiency, or severe deficiency.

	**Overall analysis**		**High Quality articles**		**Articles with high quality and Caucasian subjects**		**Articles with high quality and adjusted data**	
	***n***	**OR (95% CI)**	***n***	**OR (95% CI)**	***n***	**OR (95% CI)**	***n***	**OR (95% CI)**
Death in patients with 25OHD <10 ng/mL	21	2.60 (1.93–3.49)	13	2.61 (1.73–3.94)	8	2.95 (1.56–5.57)	8	2.27 (1.37–3.76)
Death in patients with 25OHD <20 ng/mL	24	1.84 (1.26–2.69)	13	2.54 (1.56–4.12)	8	3.57 (1.87–6.82)	4	3.37 (1.96–5.82)
Death in patients with 25OHD <30 ng/mL	12	4.15 (1.76–9.77)	7	7.06 (2.08–23.94)	3	9.57 (1.82−50.26)	2	2.18 (1.36–3.51)
ICU in patients with 25OHD <10 ng/mL	11	2.63 (1.45–4.77)	6	2.14 (1.33–3.46)	4	2.30 (0.98–5.39)	3	2.27 (0.98–5.26)
ICU in patients with 25OHD <20 ng/mL	18	2.16 (1.43–3.26)	7	2.02 (1.32–3.08)	6	2.37 (1.68–3.34)	3	2.34 (1.51–3.62)
ICU in patients with 25OHD <30 ng/mL	11	2.83 (1.74–4.61)	5	1.90 (1.27–2.84)	6	2.73 (1.19–6.27)	3	2.11 (0.72–4.85)

*ICU, intensive care unit; n, number of studies included in the analysis; OR, odds ratio*.

**Figure 4:**
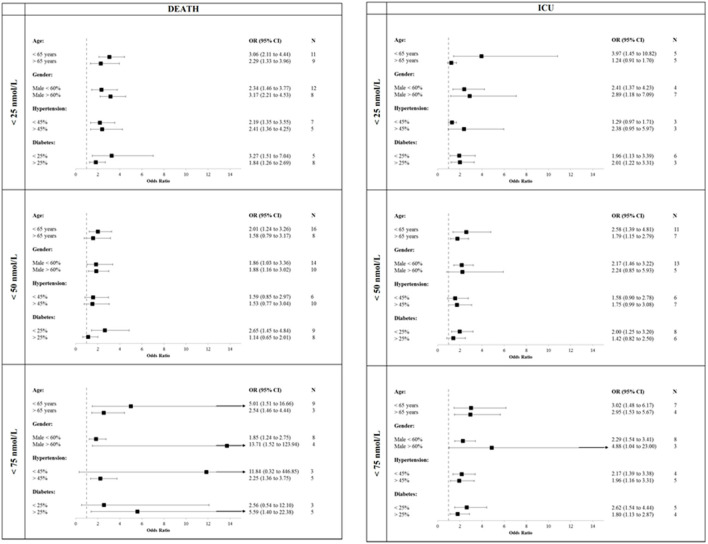
Studies stratified in categories of age, prevalence of males, hypertension and diabetes. COVID-19, Coronavirus Disease-2019; ICU, intensive Care Unit; <25 nmol/L, 25OHD levels below 25 nmol/L; <50 nmol/L, 25OHD levels below 50 nmol/L; <75 nmol/L, 25OHD levels below 75 nmol/L.

Finally, when stratifying the studies in categories of age, prevalence of males, hypertension and diabetes (Figure 4) we observed that studies reporting mean age below 65 years or high prevalence of males showed similar or slightly higher OR for all combination of primary outcomes and 25OHD levels. Inconsistent results were observed for studies stratified for both prevalence of hypertension and diabetes.

## Discussion

The primary endpoint of the present meta-analysis was to assess the association between the prevalence of vitamin D status, defined, respectively, as severe 25OHD deficiency, 25OHD deficiency or 25OHD insufficiency, and COVID-19 related severity. Overall, we found that COVID-19 cases with either vitamin D insufficiency or deficiency (including severe deficiency), assessed at hospital admission, presented an increased risk of respiratory distress (requiring ventilator support through either CPAP and/or endo-tracheal intubation and leading to ICU admission) and death from respiratory failure or other complications. The relationship between 25OHD status, assessed before SARS-CoV-2 infection, and COVID-19 severity did not reach statistical significance (except for 25OHD <75 nmol/L and ICU admission), likely due to the limited number of studies and the variable time lag between vitamin D measurement and SARS-CoV-2 infection (from 14 years to few days before diagnosis). As secondary endpoints, we also found a significant association between vitamin D status and the risk of SARS-CoV-2 infection and hospitalization. Taken together our results indicate that 25OHD is a marker of COVID-19 in-hospital severity and suggest that a good vitamin D status might have a protective role on COVID-19 clinical outcomes and even decrease the risk of becoming infected by SARS-CoV-2.

This is indeed the largest meta-analysis so far realized considering the available studies regarding the relationship between vitamin D status and COVID-19, involving more than a million individuals. Previous small metanalyses suggested a possible link between vitamin D levels and SARS-CoV-2 infection or COVID-19 infection and/or severity, but with a lower number of studies and less stringent selection criteria, thus making impossible to reach firm conclusions (17–19).

The findings of the present study, including up to 27 and 35 studies investigating the association between vitamin D status and ICU admission due to severe COVID-19 disease or related mortality, respectively, definitely indicate that, whatever the considered threshold (severe vitamin D deficiency, vitamin D deficiency, or vitamin D insufficiency), low 25OHD levels, assessed at the time of hospitalization, are associated with greater COVID-19 severity, and impact on the degree of respiratory distress (e.g., requiring ICU admission) and COVID-19 related mortality.

Several underlying pathophysiological mechanisms explaining the potential role of vitamin D against Sars-CoV-2 infection have been proposed (12). In fact, vitamin D (through its receptor) may be directly implicated in the regulation of many pathways that seem to be involved on the progression and severity of SARS-CoV-2 infection (12). As first, the effects of vitamin D on immune system may play a role (99–105). Indeed, the modulation assured by vitamin D of a possible excessive Th1 response could, in fact, contribute to counteract the cytokine storm driving to the lung damage and disease progression toward ARDS (11, 12). Moreover, vitamin D might exert a modulatory effect on neutrophil activity (106), thus reducing their excessive activation and recruitment into the inflamed lung, that is in part responsible of the alveolar damage observed in COVID-19. Likewise, a direct role of vitamin D in protecting the integrity of the pulmonary epithelial barrier and favoring epithelial repair has been suggested (107). Consistent with all these findings, vitamin D deficiency has been associated with an increased risk of developing ARDS and its correction seems to be able to reduce the alveolar capillary damage found in deficient subjects (108). This protective role of vitamin D appears to be due, in a significant proportion, to the local action of the active vitamin D metabolite, calcitriol, on the renin-angiotensin system through a direct effect on the expression of angiotensin-converting-enzymes (ACEs) (109). This observation is of particular relevance if we consider that ACE-2 is believed to be the key receptor for SARS-coronavirus infections. Finally, a good vitamin D status might counteract the pro-thrombotic state (110, 111), reducing the risk of pulmonary and systemic thrombosis that are commonly seen in severe COVID-19.

Our study has several limitations. First, in this meta-analysis of observational studies many association estimates were not adjusted. Including unadjusted associations can introduce spurious inverse associations between 25OHD levels and health outcomes due to aging, functional decline, poor general health status, obesity, comorbidities, and behavioral factors. However, as shown in Table 3, statistically significant effects were maintained when considering only high quality studies reporting adjusted estimates, both concerning mortality with all 25OHD thresholds and ICU admission with the 50 nmol/L threshold. However, these estimates were based on a reduced number of studies and we cannot exclude a potential confounding effect on pooled OR estimates.

Secondly, the fact that we have included 5 preprint studies could be questioned, since they had not been peer-reviewed at the time of the present analysis. However, for many original studies the time frame between submission and publication is unacceptable for a meta-analysis to be updated. Therefore, we have decided to include the preprint studies and then to adjust by sensitivity analysis, which did not show a relevant impact of the single study-specific association estimate on pooled odd ratios. Moreover, the present meta-analysis has included studies available until March 31th and, therefore, at the time of publication some other studies could have been published. To this regard, a website that is updated daily with the latest studies on vitamin D and COVID-19 is available at https://c19vitamind.com.

Third, many included studies derived from retrospective data and measured 25OHD levels at the time of hospital admission, thus no definitive assumptions on the causative role of a poor vitamin D status on the risk of SARS-CoV-2 infection and COVID-19 severity can be drawn. Indeed, the finding of lower 25OHD levels at SARS-CoV-2 diagnosis or at hospital admission due to COVID-19 may be expected, since 25OHD is considered an acute phase reactant and might decrease in case of severe acute infection (21, 22), via a vitamin D binding protein suppression or other mechanisms (112). The limited number of studies assessing vitamin D status before SARS-CoV-2 infection and the variable time lag occurred between 25OHD measurement and SARS-CoV-2 infection in those studies, did not allow to adequately clarify this issue, even though some of the associations remained statistically significant. This limitation could explain some discordances of previous investigations. Indeed, in a recent meta-analysis for ICU admission, inflammation, hospitalization, and pulmonary involvement, the evidence has been reported to be currently inconsistent and insufficient (19). On the other hand an updated epidemiology study on the large UK Biobank cohort (*n* = 353,299 participants with 1,082 SARS-CoV-2 affected cases) reported an association between a poor vitamin D status (albeit assessed in the baseline visit performed between 2006 and 2010) and the prevalence rates of COVID-19 hospitalization and severity (42). However, either the large time frame between 25OHD measurement and the COVID-19 infection or the possible influence of confounding factors render the results of that study questionable (113). Nevertheless, beyond its plausible causal role, the presence of vitamin D deficiency/insufficiency at hospital admission should be considered as a relevant prognostic marker of disease severity in COVID-19 patients (114).

Fourth, the high heterogeneity characterizing some specific combinations of outcome and vitamin D status, with *I*^2^ values exceeding 75% in 2/6 (33.3%) combinations in the primary endpoint and 3/6 (50%) combinations in the secondary endpoint represents an additional limitation. This heterogeneity could be particularly related to the different ethnic groups considered in the meta-analysis and to geographic and country-specific differences in the prevalence of vitamin D deficiency; this discrepancy may have affected the outcomes of the studies.

Fifth, we cannot exclude that the potential differences in both treatment protocols and criteria for SARS-CoV-2 infection diagnosis and COVID-19 hospitalization adopted by the different Countries during the pandemic flows might have affected disease outcomes and thus the results of this meta-analysis. In this respect, however, when analysis was specifically restricted to high quality studies performed in white Caucasian subjects from the European continent, all the associations between vitamin D status and the COVID-19-related primary endpoints remained statistically significant, except between severe vitamin D deficiency and ICU admission (OR 2.30, 95%CI 0.98–5.39). The latter studies were mostly performed during the first pandemic flow and in the winter season, between the 41st and 60st parallel of the Northern hemisphere, thus ruling out relevant biases due to the effects of latitude and seasonal variation on vitamin D status.

Sixth, the studies included in the present meta-analysis mostly involved patients not being stratified according to sex, BMI, age, and comorbidity. This limitation may be relevant for some findings of our analysis, since each of these parameters potentially influence both vitamin D levels and COVID-19 severity. However, similar results were observed when the meta-analysis was restricted to those studies reporting ORs adjusted for major covariates, particularly concerning COVID-19 related mortality, even if we cannot exclude residual confounding. A further attempt to stratify the results for the prevalence rates of hypertension and diabetes mellitus (being the comorbidities more frequently reported among the studies included in this meta-analysis) did not provide univocal results (Figure 4). Likewise, the presence of several, low quality studies (e.g., with a NOS score below 6) could have amplified the protective effect. However, for all 25OHD thresholds the effects reported by restricted analysis considering high quality studies were similar to those reported in the overall analysis for both the outcomes defining the primary endpoint.

Finally, an additional limitation is that different methods were used to measure serum 25OHD concentration, which can vary considerably depending on the type of assay used. Conversely, the limited number of pre-print papers included in the meta-analysis, rules out any potential bias of association.

A strength of the study is its focus on strong and well-demarked primary and secondary endpoints such as SARS-CoV-2 infection, COVID-19 related hospitalization, ICU admission and mortality in relation to the different 25OHD thresholds defining vitamin D status. While the effects of vitamin D status on either SARS-CoV-2 infection or COVID-19 related hospitalization might have been influenced by the different selection criteria of each of the involved studies (e.g., with the inclusion or exclusion of asymptomatic/mild symptomatic cases and the limited information deriving from population-based cohorts), the reported associations with disease severity and mortality strictly pertain to a more homogeneous setting of SARS-CoV-2 infected subjects with respiratory dysfunction, clearly pointing out that 25OHD is at least a relevant marker of COVID-19 severity. Furthermore, our results have a strong biological plausibility and could have a relevant impact on the clinical practice and public health. Moreover, to estimate a potential exposure-response we analyzed different 25OHD thresholds (i.e., insufficiency, deficiency and severe deficiency), allowing to a more complete definition of the risk associated with 25OHD status. While it has been impossible to identify an exact 25OHD threshold defining the risk of severe COVID-19 and death, our results suggest that 25OHD levels higher than 75 nmol/L would be required to minimize the clinical burden of SARS-CoV-2 infection, that is in line with previous observations about vitamin D status and the risk of infections in different clinical settings (115, 116).

Therefore, notwithstanding its limitations, this meta-analysis shows that at hospital admission COVID-19 patients with either vitamin D insufficiency or deficiency (including severe deficiency) present an increased risk of respiratory distress and death from respiratory failure or other complications. Moreover, subjects with a suboptimal vitamin D status seem at higher risk of SARS-CoV-2 infection, as suggested by a previous meta-analysis (117), and of COVID-19 related hospitalization. Albeit, it is not possible at this stage to confirm causality (particularly considering that many of the risk factors for severe COVID-19 outcomes are the same as the risk factors for low vitamin D status), our data are in line with the results of some recent pilot studies and meta-analysis demonstrating that a high dose of either cholecalciferol or calcidiol (25OHD) is able to reduce COVID-19 severity (14, 15, 93, 118, 119), and thus suggest that an adequate vitamin D status could be useful to contain the COVID-19 related clinical and economic burden (119, 120). In contrast with these results, a recent multicenter, double-blind, randomized, placebo-controlled trial, did not demonstrate any relevant effect of a single high dose of vitamin D on hospital length of stay or mortality rates (16). This study, however, excluded at recruitment ICU-admitted COVID-19 cases, while included a relevant number of cases testing positive for IgG against SARS-CoV-2, likely indicating an already established lung involvement (being the median time of seroconversion achieved 13 days post symptom onset) (121). Moreover, in that study vitamin D treatment started around the 11th day of symptoms. Thus, we cannot exclude that the treatment has been started too late for vitamin D to be effective (after patients have developed severe COVID-19), since the primary effects of vitamin D on SARS-CoV-2 infection are to reduce the viability of the virus through inducing cathelicidin and reducing the risk of the cytokine and chemokine storm (104).

In conclusion, the results of the present meta-analysis may be of particular relevance for subjects exposed to lockdown restrictions and especially for vulnerable groups (e.g., elderly institutionalized individuals, those with obesity or darker skin) where vitamin D deficiency is endemic. Of course, this does not diminish the importance of the ongoing vaccine campaign to counteract the health-economic burden of SARS-CoV-2 infection. Indeed, the protective effect of current vaccines is thought to possible decrease after 6–10 months and, in addition, vaccine may be less effective against new variants. Finally, also considering the safety of vitamin D supplementation (122), the achievement of an adequate vitamin D status might have a relevant role until vaccines will be widely available and, possibly, also afterwards, as an adjuvant tool to optimize the immunogenic response to vaccination.

## Data Availability Statement

The original contributions presented in the study are included in the article/Supplementary Material, further inquiries can be directed to the corresponding authors.

## Author Contributions

IC: conceptualization, data curation, funding acquisition, investigation, methodology, supervision, validation, writing—original draft, and writing—review and editing. DG: conceptualization, investigation, visualization, writing—original draft, and writing—review and editing. DS: data curation, formal analysis, investigation, methodology, resources, software, validation, visualization, writing—original draft, and writing—review and editing. DM: conceptualization, data curation, supervision, validation, visualization, and writing—original draft. CM, AFas, and GA: data curation, investigation, writing—original draft, and writing—review and editing. AFal and LP: conceptualization, supervision, validation, writing—original draft, and writing—review and editing. CE-V and MR: conceptualization, supervision, writing—original draft, and writing—review and editing. AZ: data curation, formal analysis, investigation, methodology, resources, software, validation, visualization, writing—original draft, and writing—review and editing. LG: conceptualization, data curation, formal analysis, investigation, methodology, project administration, supervision, validation, visualization, writing—original draft, and writing—review and editing. IC, LG, AZ, DG, and DS have accessed and verified the underlying data. All authors contributed to the article and approved the submitted version.

## Funding

This work was supported by a grant from Italian Ministry of Health (Ricerca Corrente Reti 2020- RCR-2020-23670065).

## Conflict of Interest

IC received speaker fees from HRA Pharma, Corcept Therapeutics, Eli-Lilly, Amgen, and UCB. DG received honoraria and/or speaker fees from Amgen, Celgene, Eli-Lilly, MSD-italia, Organon, and UCB. DM received honoraria form UCB Pharma and Savio Pharma. GA has received advisory board honoraria and consultancy fees from Theramex. CE-V has received advisory board honoraria from Kyowa Kirin, Sandoz. MR has received advisory board honoraria, consultancy fees, and/or speaker fees from Abiogen, Amgen, Abbvie, BMS, Eli-Lilly, Galapagos, Menarini, MSD, Novartis, Pfizer, Sandoz, Theramex, and UCB. LP received consultancy fees, and/or speaker fees from Merck, Sandoz, Recordati. LG received honoraria from Sandoz and Kyowa Kirin. The remaining authors declare that the research was conducted in the absence of any commercial or financial relationships that could be construed as a potential conflict of interest.

## Publisher's Note

All claims expressed in this article are solely those of the authors and do not necessarily represent those of their affiliated organizations, or those of the publisher, the editors and the reviewers. Any product that may be evaluated in this article, or claim that may be made by its manufacturer, is not guaranteed or endorsed by the publisher.

## Footnotes

^1^Available online at: https://handbook-5-1.cochrane.org/chapter_16/16_9_2_studies_with_zero_cell_counts.htm (accessed March 31, 2021).

## Supplementary Material

Supplementary Figure 1 The forest-plots reporting the association between SARS-CoV-2 infection and vitamin D thresholds (severe vitamin D deficiency, vitamin D deficiency, and vitamin D insufficiency). <25 nmol/L, 25OHD levels below 25 nmol/L; <50 nmol/L, 25OHD levels below 50 nmol/L; <75 nmol/L, 25OHD levels below 75 nmol/L.

Supplementary Figure 2 The forest-plot reporting the association between COVID-19 related hospitalization and vitamin D thresholds (severe vitamin D deficiency, vitamin D deficiency, and vitamin D insufficiency). COVID-19, Coronavirus Disease-2019; <25 nmol/L, 25OHD levels below 25 nmol/L; <50 nmol/L, 25OHD levels below 50 nmol/L; <75 nmol/L, 25OHD levels below 75 nmol/L.

Supplementary Figure 3 The funnel plots and Egger's test *p*-value for the association between admission to intensive care unit (ICU) or death and vitamin D status in COVID-19 patients. COVID-19, Coronavirus Disease-2019; ICU, intensive Care Unit; <25 nmol/L, 25OHD levels below 25 nmol/L; <50 nmol/L, 25OHD levels below 50 nmol/L; <75 nmol/L, 25OHD levels below 75 nmol/L.

Supplementary Figure 4 Influence analysis on the impact of study-specific association estimate on pooled odds ratios. ICU, intensive Care Unit; <25 nmol/L, 25OHD levels below 25 nmol/L; <50 nmol/L, 25OHD levels below 50 nmol/L; <75 nmol/L, 25OHD levels below 75 nmol/L.
