# Supplementary figures and images for: Vitamin D Status and SARS-CoV-2 Infection and COVID-19 Clinical Outcomes
Source: Front Public Health. 2021 Dec 22;9:736665. doi: 10.3389/fpubh.2021.736665 (PMC8727532; doi:10.3389/fpubh.2021.736665)

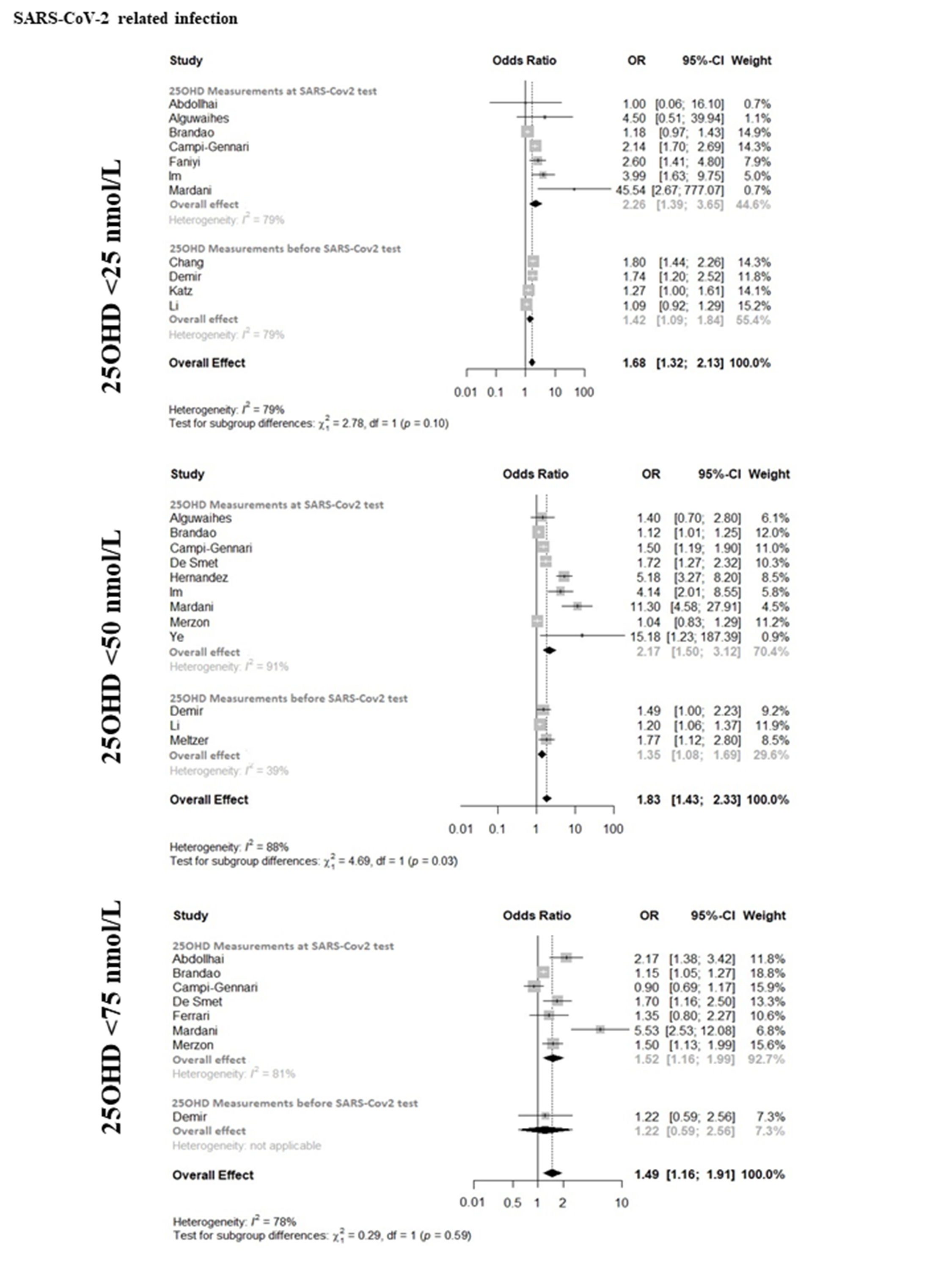

Supplement: Supplementary Figure 1 — The forest-plots reporting the association between SARS-CoV-2 infection and vitamin D thresholds (severe vitamin D deficiency, vitamin D deficiency, and vitamin D insufficiency). <25 nmol/L, 25OHD levels below 25 nmol/L; <50 nmol/L, 25OHD levels below 50 nmol/L; <75 nmol/L, 25OHD levels below 75 nmol/L. [file Image_1.TIF]

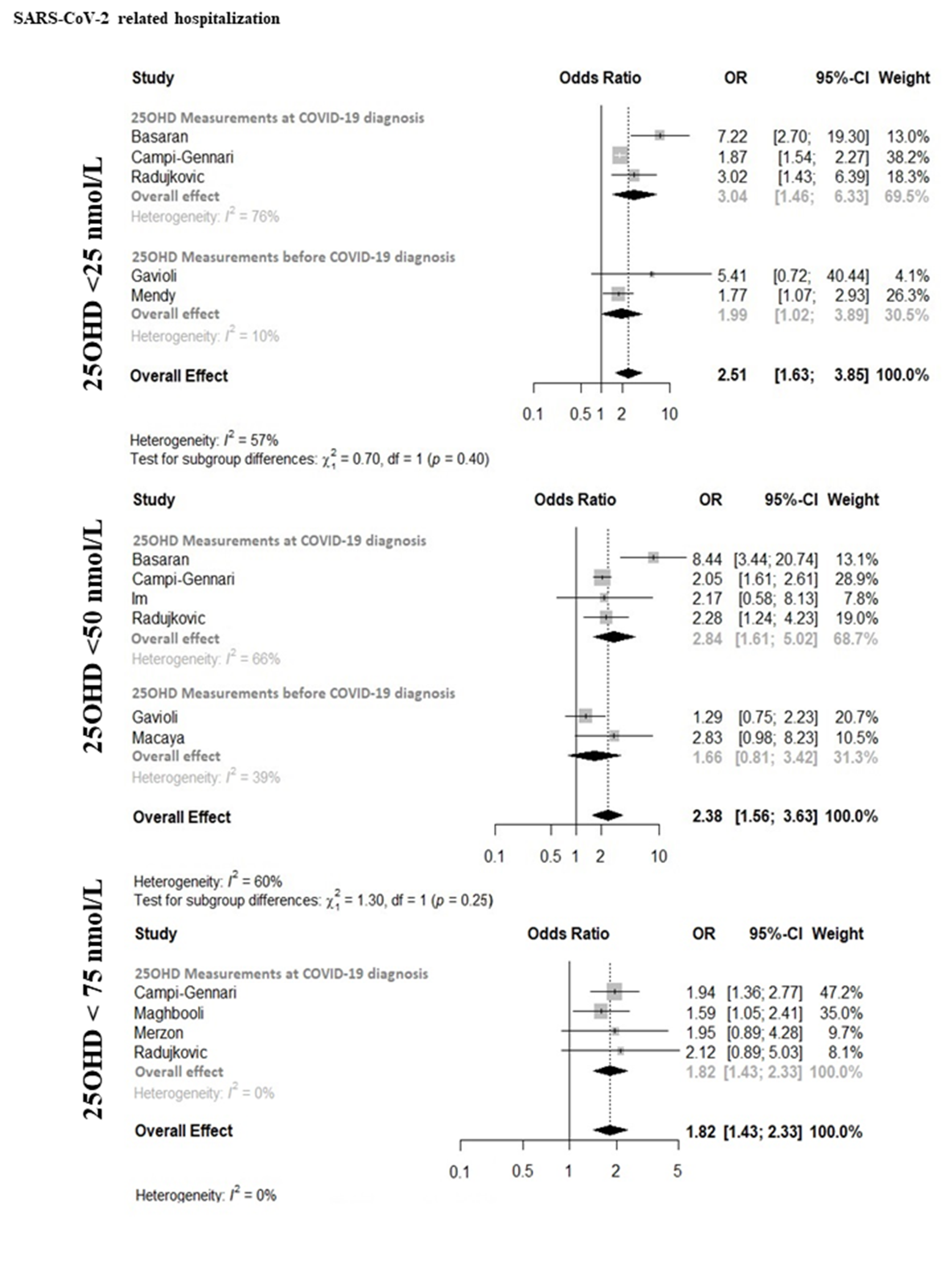

Supplement: Supplementary Figure 2 — The forest-plot reporting the association between COVID-19 related hospitalization and vitamin D thresholds (severe vitamin D deficiency, vitamin D deficiency, and vitamin D insufficiency). COVID-19, Coronavirus Disease-2019; <25 nmol/L, 25OHD levels below 25 nmol/L; <50 nmol/L, 25OHD levels below 50 nmol/L; <75 nmol/L, 25OHD levels below 75 nmol/L. [file Image_2.TIF]

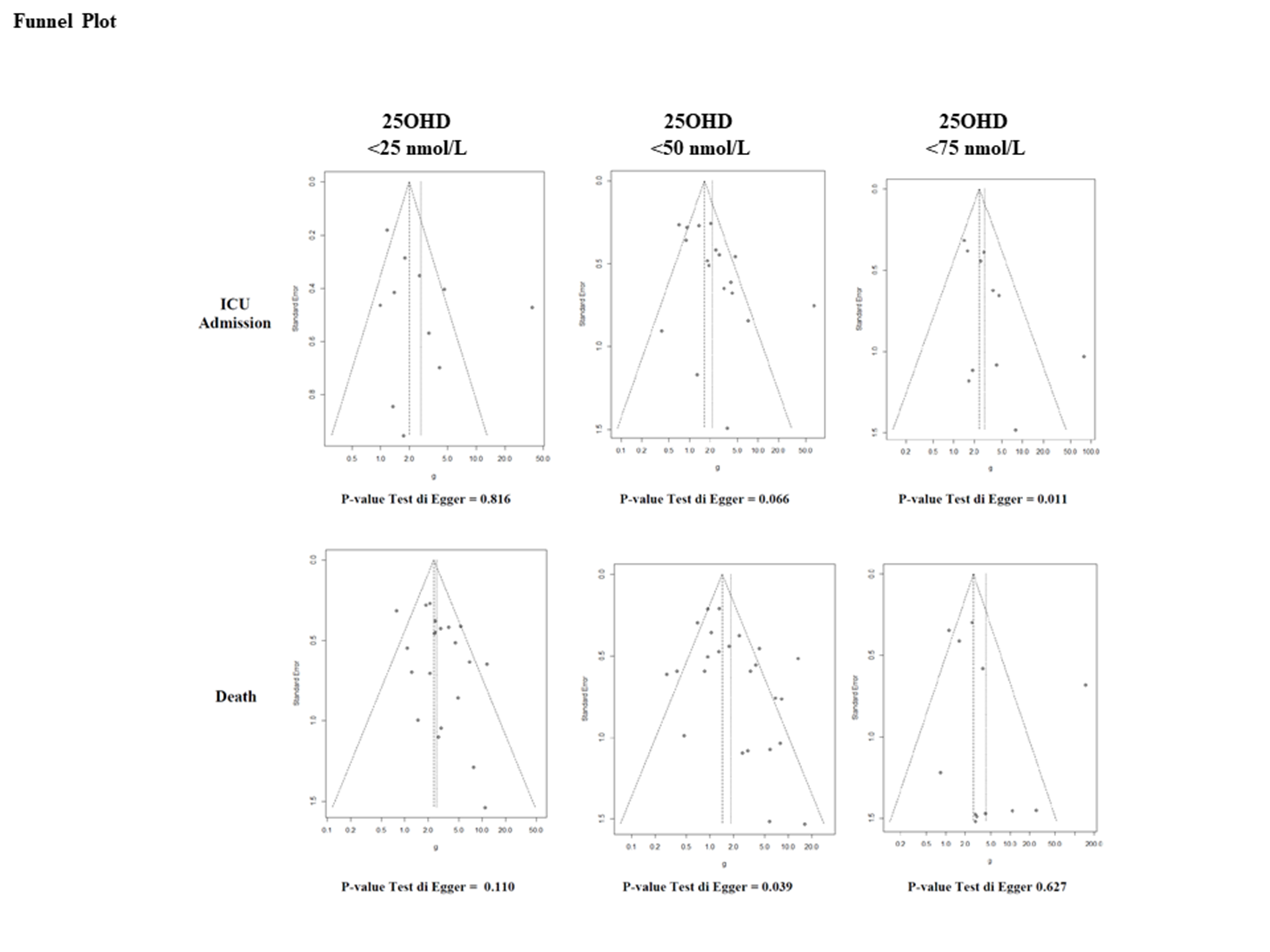

Supplement: Supplementary Figure 3 — The funnel plots and Egger's test p-value for the association between admission to intensive care unit (ICU) or death and vitamin D status in COVID-19 patients. COVID-19, Coronavirus Disease-2019; ICU, intensive Care Unit; <25 nmol/L, 25OHD levels below 25 nmol/L; <50 nmol/L, 25OHD levels below 50 nmol/L; <75 nmol/L, 25OHD levels below 75 nmol/L. [file Image_3.TIF]

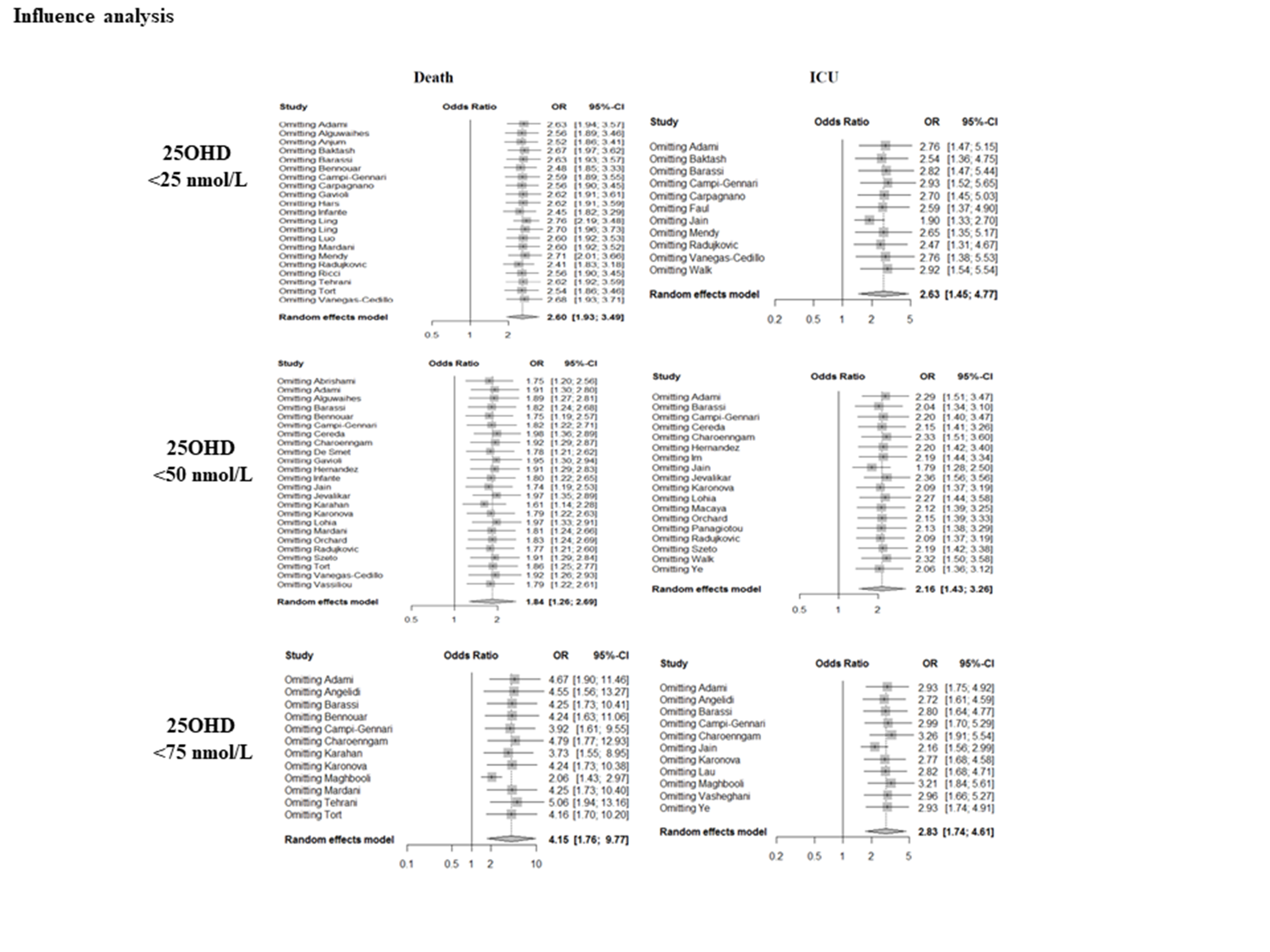

Supplement: Supplementary Figure 4 — Influence analysis on the impact of study-specific association estimate on pooled odds ratios. ICU, intensive Care Unit; <25 nmol/L, 25OHD levels below 25 nmol/L; <50 nmol/L, 25OHD levels below 50 nmol/L; <75 nmol/L, 25OHD levels below 75 nmol/L. [file Image_4.TIF]
